# Solutions to some congruence equations via suborbital graphs

**DOI:** 10.1186/s40064-016-3016-5

**Published:** 2016-08-11

**Authors:** Bahadır Özgür Güler, Tuncay Kör, Zeynep Şanlı

**Affiliations:** Department of Mathematics, Faculty of Science, Karadeniz Technical University, Trabzon, Turkey

**Keywords:** Normalizer, Imprimitive action, Suborbital graphs, 11F06, 20H10, 05C25

## Abstract

We relate the connection between the sizes of circuits in suborbital graph for the normalizer of $\Gamma _0(m)$ in PSL(2,$\mathbb {R}$) and the congruence equations arising from related group action. We give a number theoretic result which says that all prime divisors of $3u^2\mp 3u+1$ for any integer *u* must be congruent to $1\pmod {3}$.

## Background

It is known that the graph of a group provides a method by which a group can be visualized; in many cases it suggests an economical algebraic proof for a result and it gives same information but in a much more efficient way (Magnus et al. 1966). In this view, the idea of suborbital graph has been used mainly by finite group theorists.

After it was shown that this idea is also useful in the study of the modular group which is a finitely generated Fuchsian group (Jones et al. 1991), some other finitely generated groups have been studied by suborbital graphs (see Akbaş and Başkan 1996; Akbaş 2001; Akbaş et al. 2013; Beşenk et al. 2013; Deger et al. 2011; Güler et al. 2011, 2015; Kader et al. 2010; Kader and Güler 2013; Kesicioğlu et al. 2013; Keskin 2006; Kör et al. 2016). In most of them, it has been emphasized the connection between elliptic elements in group and circuits of the same order in graph closely related with the signature problem.

On the other hand, interesting number theoretic results arise from suborbital graphs as follows:

- A shortest path in subgraphs can be expressed as a continued fraction (Jones et al. 1991);
- A shortest path in trees of suborbital graphs is a special case of Pringsheim continued fraction (Deger et al. 2011);
- The subgraph $F_{1,2}$ can be defined as a new kind of continued fraction and any irrational numbers has a unique $F_{1,2}$ expansion (Sarma et al. 2015);
- The set of vertices of some suborbital graphs is strongly connected to the Fibonacci sequence (Akbaş et al. 2013).

In this light, we conclude that these graphs might be worth examining when just viewed from number theory aspect. In fact, it is well-known that modular groups have been studied much in number theory.

The aim of this paper is to examine the action of the normalizer of $\Gamma _0(m)$ which produce some congruence equations with solutions. Actually, the suborbital graphs of the normalizer were studied for some special cases (see Güler et al. 2011; Kader et al. 2010; Keskin 2006). In here, we take a different case that *m* will be of the form $3p^2$ where *p* is prime number greater than or equal to 5.

## Preliminaries

Let $PSL(2,\mathbb {R})$ denote the group of all linear fractional transformations

$$\begin{aligned} T:z \rightarrow \frac{{az + b}}{{cz + d}}, \quad \text { where }\ a,b,c\ \text { and }\ d \ \text { are real and }\ ad-bc=1. \end{aligned}$$

In terms of matrix representation, the elements of $PSL(2,\mathbb {R})$ correspond to the matrices

1 $$\begin{aligned} \pm \left( \begin{array}{cc} a &\quad b \\ c &\quad d \end{array} \right) ; \quad a,b,c,d \in \mathbb {R}\quad \mathrm {and} \quad ad-bc=1. \end{aligned}$$

This is the automorphism group of the upper half plane $\mathbb {H}:=\left\{ z\in \mathbb {C}:\mathrm {Im(z)}>0\right\}$. $\Gamma$, the modular group which is also denoted by $PSL(2,\mathbb {Z})$, is the subgroup of $PSL(2,\mathbb {R})$ such that $a,b,c\ {\text{ and }} \ d$ are integers. It is one of the most well-known and important discrete groups.

Arithmetic subgroups are finite index subgroups of the modular group. An arithmetic subgroup is said to be congruence if it contains the kernel of a modulo *m* homomorphism from $PSL(2,\mathbb {Z})$ to $PSL(2,\mathbb {Z}/m\mathbb {Z})$ for some positive integer *m*. $\Gamma _0(m)$ is the congruence subgroup of $\Gamma$ with *m*|*c*.

2 $$\begin{aligned} \mathrm {\Gamma }_0(m)={\left\{ \left( \begin{array}{cc} a &\quad b \\ c &\quad d \end{array} \right) \equiv \pm \left( \begin{array}{cc} *&\quad *\\ 0 &\quad *\end{array} \right) \pmod {m} \right\} } \end{aligned}$$

We refer the interested reader to a number of sources (Kulkarni 1991; Miyake 1989; Schoeneberg 1974; Shimura 1971) which are also useful to follow the proofs in next section.

 Conway and Norton (1977), the normalizer *Nor*(*m*) of $\Gamma _0(m)$ in $PSL(2,\mathbb {R})$ consists exactly of matrices

3 $$\begin{aligned} \left( \begin{array}{cc} {ae} & \quad {b/h} \\ {cm/h} & \quad {de} \end{array}\right) , \end{aligned}$$

where $e\parallel \frac{m}{h^2}$ and *h* is the largest divisor of 24 for which $h^2|m$ with understandings that the determinant e of the matrix is positive, and that $r\parallel s$ means that *r*|*s* and $(r,s/r)=1$ (*r* is called an exact divisor of *s*). *Nor*(*m*) is a Fuchsian group whose fundamental domain has finite area, so it has a signature consisting of the geometric invariants

4 $$\begin{aligned} (g;m_{1},\ldots ,m_{r};s) \end{aligned}$$

where *g* is the genus of the compactified quotient space, $m_{1},\ldots ,m_{r}$ are the periods of the elliptic elements and *s* is the parabolic class number.

## The action of *Nor*(*m*) on $\hat{\mathbb {Q}}$

Every element of the extended set of rationals $\hat{\mathbb {Q}}=\mathbb {Q}\cup \left\{ \infty \right\}$ can be represented as a reduced fraction $\frac{x}{y}$, with $x,y \in \mathbb {Z}$ and $(x,y)=1$; since $x/y=-x/-y$, this presentation is not unique. We represented $\infty$ as $\frac{1}{0} = \frac{-1}{0}$. The action of the matrix $$\left( \begin{array}{cc} a &\quad b \\ c &\quad d \end{array} \right)$$ on *x* / *y* is

5 $$\begin{aligned} \left( \begin{array}{cc} a &\quad b \\ c &\quad d \end{array} \right) : \frac{x}{y} \rightarrow \frac{ax+by}{cx+dy} . \end{aligned}$$

### **Lemma 1**

(Akbaş and Singerman 1992, Corollary 2) *Letm has prime power decomposition*$2^{\alpha _1}\cdot 3^{\alpha _2}\cdot p_3^{\alpha _3}\cdots p_r^{\alpha _r}$*. ThenNor(m) acts transitively on *$\hat{\mathbb {Q}}$*if and only if*$\alpha _1\le 7$*,*$\alpha _2\le 3$* and*$\alpha _i\le 1$*for*$i=3,\dots ,r$.

### **Corollary 2**

*The action of the normalizer*$Nor(3p^2)$*is not transitive on*$\hat{\mathbb {Q}}$.

### *Proof*

Since *m* is taken as the aforementioned case, the result is obvious by Lemma 1.

In this case, we will find a maximal subset of $\hat{\mathbb {Q}}$ on which the normalizer acts transitively. Since $\Gamma _0(m)\subset Nor(m)$, we give more special case before our desired result to understand the situation better. We now give a Lemma as follows.

### **Lemma 3**

(Akbaş and Başkan 1996, Theorem 4.1) *Given an arbitrary rational numberk* / *s with*$(k,s)=1$*, then there exists an element*$A\in \Gamma _0(m)$*such that*$A(k/s)=(k_1/s_1)$*with*$s_1|m$.

The following known Theorem is also proved in the same paper. We will present a different proof for the sake of completeness.

### **Lemma 4**

(Akbaş and Başkan 1996, Theorem 4.3)* Let b*|*m and let *$(a_1,b)=(a_2,b)=1$*. Then*$$\left( \begin{array}{cc} a_1 \\ b \end{array} \right)$$*and*$$\left( \begin{array}{cc} a_2 \\ b \end{array} \right)$$*are conjugate under the action of*$\Gamma _0(m)$*if and only if*$a_1\equiv a_2\pmod {t}$*, where*$t=\left( b,\frac{m}{b}\right)$.

### *Proof*

The necessary part is obvious by Lemma 3. We must prove the converse. Suppose that $a_2=a_1+t(b,m/b)$ for some $t\in \mathbb {Z}$. We need an element $$T=\left( \begin{array}{cc} k &\quad \ell \\ rm &\quad s \end{array} \right)$$ of $\Gamma _0(m)$ such that $$T\left( \begin{array}{cc} a_1 \\ b \end{array} \right) =\left( \begin{array}{cc} a_2 \\ b \end{array} \right)$$. Performing the multiplication of matrix *T* and $$\left( \begin{array}{cc} a_1 \\ b \end{array} \right)$$ we have three equations in four variables $k, \ell , r$ and *s* as follows.

$$\begin{aligned} \begin{aligned} ka_1+\ell b&=a_1+t(b,m/b)\\ ra_1\frac{m}{b}+s&=1\\ ks-rm\ell&=1. \end{aligned} \end{aligned}$$

Put $b_0=(b,m/b)$. Since $(a,b)=1$, the first equation has solutions $(k,\ell )$. So $k=\frac{a_1+tb_0-\ell b}{a_1}$ and from the second equation $s=1-ra\frac{m}{b}$. Putting these *k* and *s* into the third equation we get $r(a_1^2+atb_0)\frac{m}{bb_0}+\ell \frac{b}{b_0}=t$. The coefficient of *r* and $\frac{b}{b_0}$ are coprime. Therefore the equation has solutions *r* and $\ell$. Consequently, we have obtained an element *T* (in fact, infinitely many) in $\Gamma _0(m)$ such that $$T\left( \begin{array}{cc} a_1 \\ b \end{array} \right) =\left( \begin{array}{cc} a_2 \\ b \end{array} \right)$$.

### **Lemma 5**

(Güler et al. 2011, Corollary 2.4) *Let b*|*m. Then the orbit *$$\left( {\begin{array}{c}a\\ b\end{array}}\right)$$*of a*/*b under the action of *$\Gamma _0(m)$*is the set*$\Bigl \{x/y\in \hat{\mathbb {Q}}:(m,y)=b,a\equiv x\frac{y}{b}\pmod {\left( b,\frac{m}{b}\right) } \Bigr \}$*. Furthermore the number of orbits is*$\varphi \left( b,\frac{m}{b}\right)$*where*$\varphi (n)$* is Euler’s totient function which is the number of positive integers less than or equal to n that are coprime to n*.

### *Proof*

Lemma 3 and 4 complete the proof.

From the above we come to the following conclusion.

### **Corollary 6**

*The orbits of the action of*$\Gamma _0(3p^2)$* on*$\hat{\mathbb {Q}}$* are*

$$\begin{aligned}&\left( {\begin{array}{c}1\\ 1\end{array}}\right) ; \left( {\begin{array}{c}1\\ 3\end{array}}\right) ; \left( {\begin{array}{c}1\\ p\end{array}}\right) , \left( {\begin{array}{c}2\\ p\end{array}}\right) , \dots , \left( {\begin{array}{c}p-1\\ p\end{array}}\right) ;\\&\left( {\begin{array}{c}1\\ 3p\end{array}}\right) , \left( {\begin{array}{c}2\\ 3p\end{array}}\right) , \dots \ , \left( {\begin{array}{c}2p-1\\ 3p\end{array}}\right) ; \left( {\begin{array}{c}1\\ p^2\end{array}}\right) ; \left( {\begin{array}{c}1\\ 3p^2\end{array}}\right) . \end{aligned}$$

### *Proof*

Let us denote the representatives of the orbits by $$\left( \begin{matrix} a \\ b\end{matrix}\right)$$ as above. The possible values of *b* are $1, 3, p, 3p, p^{2}, 3p^{2}$ by Lemma 3. Hence, the number of non-conjugate classes of these orbits with Euler formula are 1 and $p-1$ for $1, 3, p^{2}, 3p^{2}$ and *p*, 3*p* respectively. By Lemma 5, the result is obvious $\square$

### **Theorem 7**

*The orbits of the action of*$Nor(3p^2)$*on*$\hat{\mathbb {Q}}$* are as follows. Let*$l\in \{1,2,\dots ,p-1\}$*. Then*

(a) If $$3\not \mid l$$*and*$$l\not \equiv p\pmod {3}$$,
  $$\begin{aligned} \left( {\begin{array}{c}l\\ p\end{array}}\right) \cup \left( {\begin{array}{c}p-l\\ p\end{array}}\right) \cup \left( {\begin{array}{c}l\\ 3p\end{array}}\right) \cup \left( {\begin{array}{c}p-l\\ 3p\end{array}}\right) \end{aligned}$$
     (b)* If*$$3\not \mid l$$* and*$$l\equiv p\pmod {3}$$,
  $$\begin{aligned} \left( {\begin{array}{c}l\\ p\end{array}}\right) \cup \left( {\begin{array}{c}p-l\\ p\end{array}}\right) \cup \left( {\begin{array}{c}l\\ 3p\end{array}}\right) \cup \left( {\begin{array}{c}2p-l\\ 3p\end{array}}\right) \end{aligned}$$
If $$3\mid l$$*, then*
  $$\begin{aligned} \left( {\begin{array}{c}l\\ p\end{array}}\right) \cup \left( {\begin{array}{c}p-l\\ p\end{array}}\right) \cup \left( {\begin{array}{c}p+l\\ 3p\end{array}}\right) \cup \left( {\begin{array}{c}p-l\\ 3p\end{array}}\right) \end{aligned}$$
  $$\begin{aligned} \left( {\begin{array}{c}1\\ 1\end{array}}\right) \cup \left( {\begin{array}{c}1\\ 3\end{array}}\right) \cup \left( {\begin{array}{c}1\\ p^2\end{array}}\right) \cup \left( {\begin{array}{c}1\\ 3p^2\end{array}}\right) \end{aligned}$$

### *Proof*

We prove only (1)-(a). The rest are done similarly. If $$T=\left( \begin{matrix} ae & \quad b \\ 3p^2c &\quad de\end{matrix}\right) \in Nor(3p^2)$$, then $e=1,3,p^2$ or $3p^2$.

(i) If $$e=1$$, then $$T\genfrac(){0.0pt}0{l}{p}=\genfrac(){0.0pt}0{l}{p}$$.
(ii) If $e=3$, then $$T\genfrac(){0.0pt}0{l}{p}=\left( \begin{matrix} 3a &\quad b \\ 3p^2c &\quad 3d\end{matrix}\right) \genfrac(){0.0pt}0{l}{p}=\frac{3al+bp}{3p^2cl+3dp}$$. Since $$det\left( \begin{matrix} 3a &\quad b \\ p^2c &\quad d\end{matrix}\right) =1$$, then $(3al+bp,p^2cl+3d)=1$. So $$\frac{3al+bp}{3p(pcl+d)}\in \left( {\begin{array}{c}x\\ 3p\end{array}}\right)$$ and $x\equiv (3al+bp)(pcl+d)\pmod {p}$. As $detT=3$, then $x\equiv l\pmod {p}$. Consequently $\genfrac(){0.0pt}0{l}{p}\cup \genfrac(){0.0pt}0{l}{3p}$.
(iii) If $e=p^2$, then $$T\genfrac(){0.0pt}0{l}{p}=\frac{apl+b}{p(3cl+dp)}\in \left( {\begin{array}{c}x\\ p\end{array}}\right)$$. As $detT=p^2$, then $x\equiv p-l\pmod {p}$. Consequently $\genfrac(){0.0pt}0{l}{p}\cup \genfrac(){0.0pt}0{p-l}{p}$.
(iv) If $e=3p^2$, then $$T\genfrac(){0.0pt}0{l}{p}=\frac{3apl+b}{3p(cl+3dp)}\in \left( {\begin{array}{c}x\\ 3p\end{array}}\right)$$. So $x\equiv -l\equiv p-l\pmod {p}$. (i), (ii), (iii) and (iv) complete the proof

$$\square$$

### **Corollary 8**

$$\hat{\mathbb {Q}}(3p^2)=\left( {\begin{array}{c}1\\ 1\end{array}}\right) \cup \left( {\begin{array}{c}1\\ 3\end{array}}\right) \cup \left( {\begin{array}{c}1\\ p^2\end{array}}\right) \cup \left( {\begin{array}{c}1\\ 3p^2\end{array}}\right)$$*is the maximal subset of*$\hat{\mathbb {Q}}$*on which the normalizer*$Nor(3p^2)$* acts transitively.*

### **Lemma 9**

*The stabilizer of a point in*$\hat{\mathbb {Q}}(3p^2)$* is an infinite cyclic group.*

### *Proof*

Because of the transitive action, stabilizers of any two points are conjugate. So it is enough to look at just $$\infty =\frac{1}{0}\in \left( {\begin{array}{c}1\\ 3p^2\end{array}}\right)$$. As $$T\genfrac(){0.0pt}0{1}{0}=\left( \begin{matrix} ae &\quad b \\ 3p^2c &\quad de\end{matrix}\right) \genfrac(){0.0pt}0{1}{0}=\frac{ae}{3p^2c}=\genfrac(){0.0pt}0{1}{0}$$, then $c=0$ and $e=1$. From the determinant equality, $$T=\left( \begin{matrix} 1 &\quad b \\ 0 &\quad 1\end{matrix}\right)$$. Consequently $$(Nor(3p^2))_\infty =\left\langle \left( \begin{matrix} 1 &\quad 1 \\ 0 &\quad 1\end{matrix}\right) \right\rangle$$.

Now we consider the imprimitivity of the action of $Nor(3p^2)$ on $\hat{\mathbb {Q}}(3p^2)$, beginning with a general discussion of primitivity of permutation groups.

Let $\left( G,\Omega \right)$ be a transitive permutation group, consisting of a group *G *acting on a set $\Omega$ transitively. An equivalence relation $\approx$ on $\Omega$ is called *G-invariant* if, whenever $\alpha ,\beta \in \Omega$ satisfy $\alpha \approx \beta$, then $g(\alpha )\approx g(\beta )$ for all $g\in G.$ The equivalence classes are called blocks.

We call $\left( G,\Omega \right)$*imprimitive* if $\Omega$ admits some *G-*invariant equivalence relation different from

(i) the identity relation, $\alpha \approx \beta$ if and only if $\alpha =\beta$;
(ii) the universal relation, $\alpha \approx \beta$ for all $\alpha ,\beta \in \Omega$.

Otherwise $\left( G,\Omega \right)$ is called *primitive*. These two relations are supposed to be trivial relations.

### **Lemma 10**

(Biggs and White 1979, Theorem 1.6.5) *Let*$\left( G,\Omega \right)$*be a transitive permutation group. Then*$\left( G,\Omega \right)$* is primitive if and only if*$G_{\alpha },$*the stabilizer of*$\alpha \in \Omega$*, is a maximal subgroup of G for each *$\alpha \in \Omega$.

From the above Lemma we see that whenever, for some $\alpha$, $G_{\alpha }\lneq H\lneq G$, then $\Omega$ admits some *G-*invariant equivalence relation other than the trivial cases. Because of the transitivity, every element of $\Omega$ has the form $g(\alpha )$ for some $g\in G$. Thus one of the non-trivial *G-*invariant equivalence relation on $\Omega$ is given as follows:

$$\begin{aligned} g(\alpha )\approx g^{\prime }(\alpha ) \hbox { if and only if } g^{\prime }\in gH. \end{aligned}$$

The number of blocks ( equivalence classes ) is the index $\left| G:H\right|$ and the block containing $\alpha$ is just the orbit $H(\alpha )$.

For applying the above to the case; let’s take that $Nor(3p^2)$, ${\hat{\mathbb {Q}}}(3p^2)$, $H_0(3p^2):=\left\langle \Gamma _0(3p^2),\genfrac(){0.0pt}0{3a \quad b}{3p^2c \quad 3d}\right\rangle$ and the stabilizer $(Nor(3p^2))_\infty$ instead of *G*, $\Omega$, *H* and $G_x$. Clearly

6 $$\begin{aligned} G_{\infty }<H_0(3p^2)<Nor(3p^2). \end{aligned}$$

### **Lemma 11**

(Akbaş and Singerman 1990, Proposition 2)* The index*$|Nor(N):\Gamma _0(N)|=2^{\rho }h^2\tau$*, where*$\rho$*is the number of prime factors of*$N/h^2$, $\tau =(\frac{3}{2})^{\varepsilon _1}(\frac{4}{3})^{\varepsilon _2}$,

$$\begin{aligned} \varepsilon _1= \left\{ \begin{array}{ll} 1 &\quad if \,2^2,2^4,2^6\parallel N \\ 0 &\quad otherwise \end{array} \right. ,\quad {\varepsilon _2= \left\{ \begin{array}{ll} 1 &\quad if \,9\parallel N \\ 0 &\quad otherwise \end{array} \right. } \end{aligned}$$

### **Theorem 12**

*The blocks arising from the imprimitive action of the normalizer by above relation (3.2) have the form:*

$$\begin{aligned}{}[0]:=\left( \begin{matrix} 1\\ 1\end{matrix}\right) \cup \left( \begin{matrix} 1\\ 3\end{matrix}\right) \quad\text {and}\quad [\infty ]:=\left( \begin{matrix} 1\\ p^{2}\end{matrix}\right) \cup \left( \begin{matrix} 1\\ 3p^{2}\end{matrix}\right) . \end{aligned}$$

### *Proof*

First, we calculate the index $|Nor(3p^2):\Gamma _0(3p^2)|$ using Lemma 11. It is clear that $h=1$ for $N=3p^2$. Furthermore, we have $\rho =2$ and $\varepsilon _1=\varepsilon _2=0$ in this case. Hence, it can be concluded that $|Nor(3p^2):\Gamma _0(3p^2)|=4$. Taking into account the definition of $H_0(3p^2)$, it is clear that $H_0(3p^2)=\Gamma _0(3p^2)\cup g\Gamma _0(3p^2)$ for the element *g* of the form $$\left( \begin{array}{cc} 3a &\quad b \\ 3p^2c &\quad 3d \end{array} \right)$$. So, we have that $|H_0(3p^2):\Gamma _0(3p^2)|=2$. Using the equation

$$\begin{aligned} |Nor(3p^2):\Gamma _0(3p^2)|= |Nor(3p^2):H_0(3p^2)|.|H_0(3p^2):\Gamma _0(3p^2)|, \end{aligned}$$

we have $|Nor(3p^2):H_0(3p^2)|=2$. So, the number of blocks is 2 by earlier comments. As we observed in Theorem 7, the orbit $\hat{\mathbb {Q}}(3p^2)$ is divided into two blocks as

$$\begin{aligned} \left( \begin{matrix} 1\\ 1\end{matrix}\right) \cup \left( \begin{matrix} 1\\ 3\end{matrix}\right) \quad\text {and}\quad \left( \begin{matrix} 1\\ p^{2}\end{matrix}\right) \cup \left( \begin{matrix} 1\\ 3p^{2}\end{matrix}\right) \end{aligned}$$

taking into account orbit $$\left( \begin{matrix} 1\\ 1\end{matrix}\right)$$ under the action of *g*.

## The suborbital graph of $Nor(3p^2)$ and $\hat{\mathbb {Q}}(3p^2)$

Sims (1967) introduced the idea of the suborbital graphs of a permutation group *G* acting on a set $\Delta$ , these are graphs with vertex-set $\Delta$, on which *G* induces automorphisms. We summarize Sims’theory as follows: Let $(G,\Delta )$ be transitive permutation group. Then *G* acts on $\Delta \times \Delta$ by $g(\alpha ,\beta )=(g(\alpha ),g(\beta )) (g\in G,\alpha ,\beta \in \Delta )$. The orbits of this action are called *suborbitals* of *G*. The orbit containing $(\alpha ,\beta )$ is denoted by $O(\alpha ,\beta )$. From $O(\alpha ,\beta )$ we can form a *suborbital graph*$G(\alpha ,\beta ):$ its vertices are the elements of $\Delta$, and there is a directed edge from $\gamma$ to $\delta$ if $(\gamma ,\delta )\in O(\alpha ,\beta )$. A directed edge from $\gamma$ to $\delta$ is denoted by $(\gamma \rightarrow \delta )$. If $(\gamma ,\delta )\in O(\alpha ,\beta )$, then we will say that there exists an edge $(\gamma \rightarrow \delta )$ in $G(\alpha ,\beta )$.

If $\alpha =\beta$, the corresponding suborbital graph $G(\alpha ,\alpha )$, called the trivial suborbital graph, is *self-paired*: it consists of a loop based at each vertex $\alpha \in \Delta$. By a *circuit* of length *m* (or a closed edge path), we mean a sequence $\nu _1 \rightarrow \nu _2 \rightarrow \dots \rightarrow \nu _m \rightarrow \nu _1$ such that $\nu _{i}\ne \nu _{j}$ for $i\ne j$, where $m\ge 3$. If $m=3, 4$ and 6, then the circuit is called a triangle, a quadrilateral and a hexagon, respectively.

We now investigate the suborbital graphs for the action of $Nor(3p^2)$ on $\hat{\mathbb {Q}}(3p^2)$. Since the action of $Nor(3p^2)$ on $\hat{\mathbb {Q}}(3p^2)$ is transitive, $Nor(3p^2)$ permutes the blocks transitively; so the subgraphs are all isomorphic. Hence it is sufficent to study with only one block. On the other hand, it is clear that each non-trivial suborbital graph contains a pair ($\infty ,u/p^2$) for some $u/p^2\in \hat{\mathbb {Q}}(3p^2)$. We let $F(\infty ,u/p^2)$ be the subgraph of $G(\infty ,u/p^2)$ whose vertices form the block $$[\infty ]=\left( \begin{matrix} 1\\ p^{2}\end{matrix}\right) \cup \left( \begin{matrix} 1\\ 3p^{2}\end{matrix}\right)$$, so that $G(\infty ,u/p^2)$ consists of two disjoint copies of $F(\infty ,u/p^2)$.

### **Theorem 13**

(Edge condition)* Letr*/*s* and *x*/*y be in the block *$[\infty ]$*. Then there is an edge*$r/s\rightarrow x/y$*in*$F(\infty ,u/p^2)$* if and only if*

(i) If $p^2|s$ but $3p^2\not \mid s$, then $x\equiv \mp 3ur\pmod {p^2}, y\equiv \mp 3us\pmod {3p^2}$, $ry-sx=\mp p^2$
(ii) If $3p^2|s$ then $x\equiv \mp ur\pmod {p^2}, y\equiv \mp us\pmod {p^2}, ry-sx=\mp p^2$

### *Proof*

Assume first $\frac{r}{s} \rightarrow \frac{x}{y}$ is an edge in $F(\infty ,u/p^2)$ and $p^2|s$ but $3p^2\not \mid s$. Therefore there exists some *T* in the normalizer $Nor(3p^2)$ such that *T* sends the pair $(\infty , u/p^2)$ to the pair (*r* / *s*, *x* / *y*), that is $T(\infty )=r/s$ and $T(u/p^2)=x/y$. Since $3p^2\not \mid s$, *T* must be of the form $\genfrac(){0.0pt}0{3a \qquad b}{3p^2c \quad 3d}$. $T(\infty ) = 3a/3p^2c = \genfrac(){0.0pt}0{(-1)^ir}{(-1)^is}$ gives that $r=(-1)^ia$ and $s=(-1)^ip^2c$, for $i=0,1$. $$T(u/p^2) =\left( \begin{matrix} 3a & \quad b \\ 3p^2c &\quad 3d\end{matrix}\right) \genfrac(){0.0pt}0{u}{p^2}=$$

$$\begin{aligned} = \left( \begin{array}{c} 3au+bp^2\\ 3p^2cu+3dp^2 \end{array} \right) = \left( \begin{array}{c} (-1)^jx \\ (-1)^jy \end{array} \right) \quad \mathrm{for} \ j=0,1. \end{aligned}$$

Since the matrix $\genfrac(){0.0pt}0{3a \quad b}{p^2c \quad d}$ has determinant 1 and $(u,p^2)=1$, then $(3au+bp^2, p^2cu+dp^2)=1$. Therefore $(3au+bp^2, 3p^2cu+3dp^2)=1$. So

$$\begin{aligned} x = (-1)^j(3au+bp^2) , \ y = (-1)^j(3p^2cu+3dp^2) . \end{aligned}$$

That is, $x=(-1)^{i+j}3au\pmod {p^2}$, $y=(-1)^{i+j}3su\pmod {3p^2}$. Finally, since

$$\begin{aligned} \left( \begin{array}{cc} 3a & \quad b\\ 3p^2c & \quad 3d \end{array} \right) \left( \begin{array}{cc} 1 &\quad u\\ 0 &\quad p^2 \end{array} \right) = \left( \begin{array}{cc} (-1)^i3r &\quad (-1)^jx \\ (-1)^i3s &\quad (-1)^jy \end{array} \right) \quad \mathrm{for} \ i,j=0,1, \end{aligned}$$

we get $ry-sx=\mp p^2$. This proves (i).

Secondly let $\frac{r}{s} \rightarrow \frac{x}{y}$ be an edge in $F(\infty ,u/p^2)$ and $3p^2|s$. In this case *T* must be of the form $\genfrac(){0.0pt}0{a \quad b}{3p^2c \ d}$, $\det T=1$. Therefore, since $T(\infty ) = \genfrac(){0.0pt}0{a}{3p^2c} = \genfrac(){0.0pt}0{(-1)^ir}{(-1)^is}$ we get $a=r$ and $s=3p^2c$, by taking *i* to be 0. Likewise, since

$$\begin{aligned} \left( \begin{array}{cc} a &\quad b\\ 3p^2c &\quad d \end{array} \right) \left( \begin{array}{c} u\\ p^2 \end{array} \right) = \left( \begin{array}{c} au+bp^2\\ 3p^2cu+dp^2 \end{array} \right) = \left( \begin{array}{c} (-1)^jx \\ (-1)^jy \end{array} \right) , \end{aligned}$$

we have $x\equiv ur$(mod $p^2$) and $y\equiv us$(mod $p^2$) and that $ry-sx=p^2$ with $j=0$. The case where $i=0$ and $j=1$ gives (b).

In the opposite direction we do calculations only for (i)(a). The others are likewise done. So suppose $x\equiv 3ur\pmod {p^2}$, $y\equiv 3us\pmod {3p^2}$, $ry-sx=p^2$, $p^2|s$ and $3p^2\not \mid s$. Therefore there exist *b*, *d* in $\mathbb {Z}$ such that $x=3ur+p^2b$ and $y=3su+3p^2d$. Since $ry-sx=p^2$, we get $3rd-bs=1$, or $9rd-3bs=3$. Hence the element $T:=\genfrac(){0.0pt}0{3r \quad b}{3s \quad 3d}$ is not only in the normalizer $Nor(3p^2)$, but also in *H*. It is obvious that $T(\infty )=\genfrac(){0.0pt}0{r}{s}$ and $T\genfrac(){0.0pt}0{u}{p^2}=\genfrac(){0.0pt}0{x}{y}$. $\square$

### Farey graph and subgraph $F(\infty ,u/p^2)$

Now, let us represent the edges of $F(\infty ,u/p^2)$ as hyperbolic geodesics in the upper half-plane $\mathbb {H}$, that is, as Euclidean semi-circles or half-lines perpendicular to real line as in Jones and Singerman (1987). To understand the situation better, we give the Farey graph and some its properties as follows:

#### **Definition 14**

**Fig. 1:**
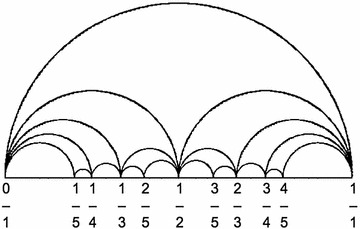
Farey graph

The Farey graph, denoted by F, is defined as : the vertex $\infty$ is joined to the integers, while two rational numbers *r/s* and *x*/*y* (in reduced form) are adjacent in F if and only if $r/s-x/y=\mp 1$, or equivalently if they are consecutive terms in some Farey sequence $F_{m}$ (consisting of the rationals x/*y with*$|y|\le m$, arranged in increasing order). See also Fig. 1.

#### **Lemma 15**

(Jones et al. 1991, Corollary 4.2)* No edges ofF cross in*$\mathbb {H}$.

Similar result can be given by both of following useful Lemma and Theorem 13 as in (Jones et al. 1991);

#### **Lemma 16**

*Let r*/*s and x*/*y be rational numbers such that*$r/s-x/y=-1$*, where*$s\ge 1$, $y\ge 1$*. Then there exist no integers between r*/*s and x*/*y*.

#### *Proof*

Let *k* be an integer such that $r/s<k<x/y$. Then $r<sk$ and $x>ky$. Thus $1=sx-ry>sx-sky=s(x-ky)\ge s$, which is a contradiction.

#### **Theorem 17**

*No edges of the subgraph *$F(\infty ,u/p^2)$*of*$Nor(3p^2)$*cross in*$\mathbb {H}$.

#### *Proof*

Without loss of generality, because of the transitive action, we can take the edges $\infty \rightarrow \frac{u}{p^2}$, $\frac{x_1}{y_1p^2}\rightarrow \frac{x_2}{y_2p^2}$ and $\frac{x_1}{y_1p^2}<\frac{u}{p^2}< \frac{x_2}{y_2p^2}$, where all letters are positive integers. It is easily seen that $x_1y_2p^2-x_2y_1p^2=-p^2$ by Theorem 13. $\frac{x_1}{y_1}<u< \frac{x_2}{y_2}$ and Lemma 16 complete the proof.

## Results

### **Theorem 18**

$F(\infty ,u/p^2)$*has a self-paired edge iff*$3u^2\equiv -1\pmod {p^2}$.

### *Proof*

Because of the transitive action, the form of self-paired edge can be taken of $1/0\rightarrow u/p^2\rightarrow 1/0$. The condition follows immediately from the second edge by Theorem 13.

### **Theorem 19**

$F(\infty ,u/p^2)$*has no triangle or quadrilateral.*

### *Proof*

We suppose that it has a triangle. Because of the transitive action, it must be of the form $1/0\rightarrow u/p^2\rightarrow x/3p^2y\rightarrow 1/0$. But this contradicts Theorem 13 which says that both denominators of vertices of an edge having the form $\frac{r}{s}\rightarrow \frac{x}{y}$ are not divisible by 3 at the same time. We now suppose that it has a quadrilateral. It must be of the form $1/0\rightarrow u/p^2\rightarrow x/3p^2y\rightarrow k/p^2\rightarrow 1/0$ by same reason. From second, third and fourth edges by Theorem 13, we have the equations; $3uy-x=-1$, $x-3ky=-1$ and $1\equiv -3uk\pmod {p^2}$. Therefore we obtain a contradiction 3|2.

### **Theorem 20**

*If*$3u^2\mp 3u+1 \equiv 0\pmod {p^2}$, $F(\infty ,u/p^2)$* has a hexagon.*

### *Proof*

By Theorem 13, we obtain easily that

$$\begin{aligned} \infty \rightarrow \frac{u}{p^2}\rightarrow \frac{3u\mp 1}{3p^2}\rightarrow \frac{2u\mp 1}{2p^2}\rightarrow \frac{3u\mp 2}{3p^2} \rightarrow \frac{u\mp 1}{p^2}\rightarrow \infty \end{aligned}$$

**Fig. 2:**
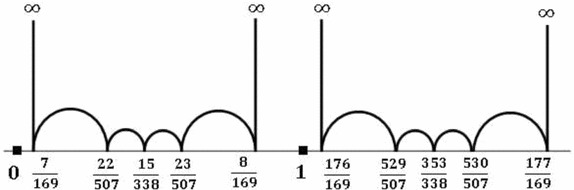
Subgraph $$F(\infty ,7/169)$$

As an example, we can verify easily $\frac{1}{0}\rightarrow \frac{7}{169}\rightarrow \frac{22}{507}\rightarrow \frac{15}{338}\rightarrow \frac{23}{507}\rightarrow \frac{8}{169}\rightarrow \frac{1}{0}$ is a hexagon in $F(\infty ,7/169)$. See also Fig. 2.

### **Theorem 21**

$H_0(3p^2)$*contains an elliptic element*$\varphi$*of order 6 if and only if*$F(\infty ,u/p^2)$*contains a hexagon.*

### *Proof*

Taking into account (3), we suppose that $$\varphi =\left( \begin{array}{cc} {3a} &\quad {b} \\ {3p^2c} &\quad {3d} \end{array}\right)$$ is an elliptic element of order 6. It is known that $a+d=\pm 1$ for order 3, 4, 6. Since $det=3$, we have $3a(\pm 1-a)\equiv 1$(mod$p^2$), that is $3a^2\mp 3a+1\equiv 1$(mod$p^2$). As $(a,n)=1$, $F(\infty ,u/p^2)$ contains a hexagon by above Theorem.

Conversely, we suppose that $F(\infty ,u/p^2)$ contains a hexagon. Because of the transitive action, we have

$$\begin{aligned} \infty \rightarrow \frac{u}{p^2}\rightarrow \frac{3u\mp 1}{3p^2}\rightarrow \frac{2u\mp 1}{2p^2}\rightarrow \frac{3u\mp 2}{3p^2} \rightarrow \frac{u\mp 1}{p^2}\rightarrow \infty \end{aligned}$$

Hence we get the element $$\varphi :=\left( \begin{array}{cc} -3u &\quad (3u^2\mp 3u+1)/p^2 \\ -3p^2 &\quad 3u+3 \end{array} \right)$$.

### **Lemma 22**

(Akbaş and Singerman 1990, Theorem 2) *The periods of elliptic elements of Nor(m)may be 2, 3, 4, 6. Nor(m) has at most one period of order 6. It has a period of order 6 iff *$3\Vert m/h^2$* and if p is an odd prime divisor of *$m/h^2$*then*$p\equiv 1\pmod {3}$.

### **Corollary 23**

*The prime divisors p of*$3u^2\mp 3u+1$*, for any*$u\in \mathbb {Z}$*, are of the form*$p\equiv 1\pmod {3}$.

### *Proof*

Let *p* a prime number and a divisor of $3u^2\mp 3u+1$ for any integer *u*. In this case, it is clear that *Nor*(3*p*) has the elliptic element $$\left( \begin{array}{cc} -3u &\quad (3u^2\mp 3u+1)/p \\ -3p &\quad 3u+3\end{array} \right)$$ of order 6 as in $Nor(3p^2)$. We get $p\equiv 1\pmod {3}$ by above Lemma.

## Conclusions

Because this work combine different fields of mathematics such as algebra, geometry, group theory and number theory, it can be seen as an example of multidisciplinary approach which offer a new understanding of some situations. We show that we can produce solutions for some number theoretic problems using finite group theory once again. Taking into account the conjecture (Güler et al. 2011) which is also confirmed for the simplest hexagonal case within non-transitive cases by this paper, the normalizer has a potential to suggest solutions for other congruence equations such as $8u^2\mp 4u+1\equiv 0\pmod {p}$, $9u^2\mp 3u+1\equiv 0\pmod {p}$, $27u^2\mp 9u+1\equiv 0\pmod {p}$ etc.
